# Are youth mentoring programs good value-for-money? An evaluation of the Big Brothers Big Sisters Melbourne Program

**DOI:** 10.1186/1471-2458-9-41

**Published:** 2009-01-30

**Authors:** Marjory L Moodie, Jane Fisher

**Affiliations:** 1 Health Economics Unit, Public Health Research Evaluation and Policy Cluster, Deakin University, 221 Burwood Highway, Burwood, Victoria 3125, Australia; 2 The Key Centre for Women's Health in Society, School of Population Health, The University of Melbourne, Victoria 3010, Australia

## Abstract

**Background:**

The Big Brothers Big Sisters (BBBS) program matches vulnerable young people with a trained, supervised adult volunteer as mentor. The young people are typically seriously disadvantaged, with multiple psychosocial problems.

**Methods:**

Threshold analysis was undertaken to determine whether investment in the program was a worthwhile use of limited public funds. The potential cost savings were based on US estimates of life-time costs associated with high-risk youth who drop out-of-school and become adult criminals. The intervention was modelled for children aged 10–14 years residing in Melbourne in 2004.

**Results:**

If the program serviced 2,208 of the most vulnerable young people, it would cost AUD 39.5 M. Assuming 50% were high-risk, the associated costs of their adult criminality would be AUD 3.3 billion. To break even, the program would need to avert high-risk behaviours in only 1.3% (14/1,104) of participants.

**Conclusion:**

This indicative evaluation suggests that the BBBS program represents excellent 'value for money'.

## Background

Mentoring involves the commitment of time and specific efforts by a more experienced person to the development of a mutually beneficial, supportive and nurturing relationship with a less experienced person. Big Brothers Big Sisters (BBBS) is a world wide organisation which has operated in Melbourne (BBBS-M), Australia since 1980. Its program matches vulnerable young people (termed "Littles"), who are aged between 7 and 17 years, living in complex social predicaments, are isolated and in need of additional support and friendship, with adult volunteers (termed "Big Brothers" or " Big Sisters" or "Bigs"). The program aims to reduce marginalisation and/or self-destructive behaviours in young people and to foster optimal development and the establishment of confident adult identities.

As at November 2003, there were 439 Littles on the database of BBBS-M. Of these, 109 were in active matches with a Big, whilst a further 189 were on the waiting list for a match, and 141 were classified as 'non-active' (most of whom had been in a match which had been completed, the majority through a process of graduation) (see Table 1) [1]. There were more than twice as many girls than boys actively matched, and there were more boys on the waiting list. Most Littles (58%) were aged 10 years or under at the time of referral, whilst a further 36% were early adolescents (11–14 years). Boys were more likely than girls to be referred at a younger age, whereas girls predominated amongst the adolescent referrals. There were diverse reasons for referral to the program, but overall the client group was seriously disadvantaged. Common reasons for referral include problematic social behaviour or conduct disturbance; exposure to parental conflict including domestic violence; court ordered child protection for risk of abuse; parental psychiatric illness or substance abuse; having a sibling with a disability or living in foster family or residential care. All lacked suitable role models and were experiencing marginalisation or social isolation [1].

**Table 1:** Profile of Littles and Bigs in BBBS-M program (as at November 2003)

**Variable**		**Current**	**Past**	**Waiting**	**Rejected**	**Total**
**Littles**	Number	109	141	189		439
						
		%	%	%	%	%
Gender	Males	31	56	56		49
	Females	70	44	44		51
						
Age at referral	5–7 years	24	19	17		19
	8–10 years	47	40	32		38
	11–14 years	26	33	43		36
	15–18 years	3	4	5		4
	Not available		4	2		2
						
Primary reason for referral	Lack of role model					42
	Isolation					17
	Parental substance abuse					2
	Parental psychiatric disability					7
	Sibling disability					3
	Abuse					4
	Lack of peer friendships					3
	Other					22
						
**Bigs**	Number	107	148	24	23	302
		%	%	%	%	%
						
Gender	Males	31	30	8	39	30
	Females	69	70	92	61	70
						
Age at referral	<20 years	6	8	4	4	7
	20–29 years	47	51	46	30	47
	30–39 years	37	32	46	26	35
	40 years and over	10	7	4	35	11
	Not available		1		4	
						
Marital status	Single	66	70	67	61	68
	Married/de facto	25	19	21	13	21
	Divorced	5	5	8	4	5
	Other/not available	4	7	4	22	6

Most Bigs were single, professional or managerial workers, aged in their twenties, and there were twice as many females than males (Table 1). Most closed matches (74%) had continued beyond the required initial commitment of twelve months' duration. Thirty per cent had continued for between 2 and 5 years and 17% for between 5 and 10 years (see Table 2). Of the matches still current at November 2003, 64% had lasted more than one year. A change in circumstance, most usually of the Big (for example, an interstate move for professional reasons), was the most common reason for premature termination of a match, with very few matches terminating early because of dissatisfaction of either or both parties [1].

**Table 2:** Length of matches by gender and current status, BBBS-M program (as at November 2003)

**Length of match** **(months)**	**Current** **(n = 110)** **%**	**Completed** **(n = 94)** **%**	**Total** **(n = 204)** **%**
0 – 12 (< one year)	36	25	31
13 – 24 (one – two years)	26	27	27
25 – 36 (two-three years)	16	11	13
37 – 60 (three – five years)	11	19	15
61 – 120 (five – ten years)	11	17	13
Not available		1	1
Total	100	100	100

Length of contact between mentors and youth has been identified as a key indicator of the likely effectiveness of mentoring. Mentoring relationships which are sustained over a significant period of time are more likely to lead to beneficial outcomes [2,3], and are likely to be increasingly effective with time. However, whilst the BBBS-M program performs well in this regard and is highly regarded by stakeholders, its sustained funding requires more rigorous demonstration of its effectiveness and cost-effectiveness.

Mentoring has been widely promoted as a mechanism for helping vulnerable young people. Whilst there has been a proliferation of programs, both in Australia and overseas, which provide mentoring for young people at-risk of short or long term psychological and social problems, very few have been evaluated [4]. Roberts, 2004 [5] equated the effectiveness of mentoring, on the basis of current knowledge, to that of a new drug in that it "shows promise but remains in need of further research and development". The question posed by a number of authors [6] is whether young people who participate in these programs are better off as a result of their participation.

### Economic evaluations of mentoring for "at-risk" youth

Few mentoring programs for young people "at-risk" have been rigorously evaluated in terms of their effectiveness in promoting maturity and positive social adjustment in young people and there is a dearth of evidence about their cost-effectiveness. Grossman & Furano, 1999 [7] note that "*while it (mentoring) appears to be relatively inexpensive for a social policy intervention....... little is known about the cost of mentoring and how this relates to program features*".

Fountain & Arbreton, 1999 [8] endeavoured to overcome the lack of data on program costs by sampling 52 different mentoring programs. They highlighted the difficulty of ascertaining cost measurement accurately because most mentoring programs rely on donated resources in addition to or outside the funded budget. The study found that mentoring programs leveraged the equivalent of about $1 for every $1 in their budget. The average program budget (across the 52 programs) of AUD 262,651 (converted from US dollar values using OECD purchasing power parities) was augmented by donated goods and services (including volunteer time) to the value of AUD 267,515, giving a total average program cost of AUD 530,165. In a given year, the average program spent a total cost of AUD 1,856 per young person served (median AUD 1,248).

A number of studies have examined the relationship between the effectiveness and costs of youth mentoring programs. A rate of return analysis of BBBS-America conducted by Belfield, 2003 [9] considered the program as an investment in education and drew on the impact evaluation of 1,148 young people randomly assigned to the BBBS-America program or a waiting list control group [10,11]. Belfield concluded that the program yielded a positive rate of return to both the youth participants, the mentors and to society as a whole, as a consequence of the participants' increased earning potential and reduced negative behaviours.

A cost-benefit analysis of prevention and early intervention programs for at-risk youth conducted by the Washington State Institute for Public Policy 2004 [12] concluded that some well-implemented programs achieved significantly more benefits than costs. With specific reference to BBBS-A, the study reported that the benefits and costs of the program per youth were US4,058 and US4010 (and US1236 if no monetary value is placed on volunteer time) respectively, resulting in a small benefit per dollar cost of US1.01 (or US3.28 to the taxpayer only). A more recent study [13] showed that a youth mentoring program returned benefits of US2.72 for every dollar of resources invested.

In order to begin to build an evidence base to practice, the aim of our study was to prepare an economic case for the public worth of the BBBS-M program, by investigating whether the current program represents 'value for money'. The specific objective was to establish whether an investment in the program is likely to be a worthwhile use of limited public health and welfare resources? In establishing the economic credentials of the program, the focus is on allocative efficiency.

## Methods

### The intervention

The BBBS-M program offers formal supervised mentoring on a one-on-one basis as the sole focus of a stand-alone program, in contrast to other programs where mentoring is offered on a group basis or where it is one of multiple activities within an integrated youth program [4].

### Study parameters

#### (i) Study design

To ascertain whether the program provides 'value for money", economic evaluation using "threshold analysis" [14] was used. The latter is a technique which can be used to assist resource allocation decisions, by identifying the critical values of parameters underpinning decisions to invest in the program. It determines the threshold with regard to costs and effects (both measured in monetary terms) that the intervention must achieve to be acceptable. So the task was to assess at what point is the BBBS-M program likely to 'break even' in cost terms. More specifically, at what point are any cost-offsets, defined as savings arising from the reduction in the marginalisation/self-destructive behaviour of youth as a result of the program greater than the costs of implementing the program.

#### (ii) Study perspective

It is important to specify the viewpoint of the economic evaluation since an item may be a cost from one point of view, but not from another [14]. A traditional health sector perspective as employed in many economic evaluations was not appropriate as the major impact of negative behaviours targeted by the program occur in sectors other than health (such as education, social welfare and criminal justice). A broader social perspective was assumed.

#### (iii) Study comparator

The comparator was a scenario in which participants did not receive the BBBS-M intervention.

#### (iv) Reference year

The reference year for the evaluation was the calendar year ending June 2004.

#### (v) Study population

The population of interest was young people living in the Melbourne metropolitan area aged 10–14 years, which is within the age range for eligibility for the BBBS-M program.

### Identification, measurement and valuation of costs

#### (i) Costs of the intervention

The costs included all the resource use associated with the delivery of the program during the reference year. The costs of BBBS-M include labour, office accommodation and associated costs, transport and other administrative infrastructure costs (such as telephone and printing) (see Additional file 1). The unit costs and resource utilisation were primarily sourced from BBBS-M financial records.

The program uses a range of resources for which it incurs no costs, examples of which are 'pro bono' legal and public relations advice and administrative assistance; free rental accommodation, and the time which Bigs donate to the program. Given that this evaluation is concerned to identify the real economic costs associated with the operating the service, the costing included both 'budgeted' and 'off-budget' items, with market values being imputed for the latter. Mentors are required to commit to between two to six hours per week of contact with their Little during the first year of the match. Time is an important resource, and has an opportunity cost (both in terms of production effects and the intrinsic value of time *per se*). For this reason, volunteer time was valued at AUD16 per hour, which is the average hourly rate paid by other Australian mentoring programs which pay mentors a stipend [4].

Whilst costs incurred by Bigs (time, travel, telephone) were factored into the costing, no costs to Littles and their families were included. Young people actively matched with a mentor are likely to incur limited costs as a result of their participation in the program. Any expenses associated with contact visits with their mentors are likely to be minor and are usually borne by the Big. These expenses were not included. Likewise, the time foregone by Littles to other activities as a result of their participation in the program was not costed.

In calculating the full economic cost of running the BBBS-M program in 2003–04, the program was assumed to be operating in 'steady state' or at its full effectiveness with no workforce or unmet training needs. It was assumed that an appropriate workforce was available to deliver the intervention to the recruited population, meaning that no provision was made for costs associated with the recruitment, orientation and training of more staff.

#### (ii) Costs associated with the marginalisation and risk behaviours of youth

The potential benefits of youth mentoring programs include gains of a diverse nature [6,15,16], ranging from improvements in academic performance [17-19], decreased involvement in unhealthy or unsafe activities such as drug or alcohol use [10], early sexual debut or risky sexual behaviours and teenage pregnancies [20] and antisocial behaviour and juvenile crime [10,19,21]. Rather than taking such a multi-dimensional approach, this evaluation focused on cost-offsets to society through any reduction in crime (juvenile and adult) as a result of participation in the program. This focus reflects the Australian government interest in the measurement of outcomes of mentoring with respect to juvenile crime and the criminal justice system [4].

A search of the published literature for Australian data about the costs of marginalised youth who become involved in crime identified a number of studies which report unit cost estimates per different categories of crime [22-24]. The latter [24] is the most comprehensive, however it does not attempt to calculate the costs of a typical life of crime, and also does not include some major cost components such as lost productivity of prisoners. A report by the NSW Attorney-General's Department (n.d.) [25] indicated that recorded juvenile crime is concentrated at the 'less serious' end of the spectrum, and that the financial costs (in terms of property or other losses) are relatively small in comparison to adult crime. Nevertheless, the personal costs stemming from anger, fear, anxiety and emotional distress of the victims are likely to be serious and substantial.

Of more relevance for the purposes of this investigation is a US study by Cohen, 1998 [26] which set out to determine the potential benefits from 'saving' a high-risk youth. Using a 2% discount rate, the study found that the typical career criminal causes AUD 1.98–2.2 M in external costs, a heavy drug user AUD 0.6–1.48 M and a high-school dropout AUD 0.4–0.6 M over their lifetime. Eliminating duplication between crimes committed by individuals who are both heavy drug users and career criminals, Cohen concluded that the overall estimate of the 'monetary value of saving [one] high-risk youth' was of the order of AUD 2.6–3.5 M. Whilst acknowledging that the estimates are subject to considerable uncertainty, the author believed that they were reasonable and substantiated with real-world data, and used sensitivity analysis to test the effect of varying the assumptions. The study used a target population of 'chronic juvenile offenders' and drew on estimates of annual rates of offending and typical length of criminal careers [27] and of time spent in prison [28]. The lifetime cost estimates took into account tangible victim costs, lost quality of life, medical and treatment costs, criminal justice costs and offender productivity losses. In our research, we therefore attached a monetary value of AUD 3 M to the saving of one high-risk youth.

The Cohen costs [26] were converted from 1997 US dollars to 2002–03 Australian dollars using a two stage procedure. Firstly, the US costs were converted to 1997 AUD using purchasing power parities . These values were inflated to 2002–03 equivalent values using the relevant Australian health price deflator. It was assumed that the benefits started to apply at age 18 years; they were then discounted at 5% back to age 10 years (the age of many children in the program) to be consistent with costs.

### Identification, measurement and valuation of benefits

The measurement of the costs-offsets arising from the BBBS-M program averting young people from a life of crime required evidence about the probability of children in the program target group following such a pathway, and secondly, the likelihood of such activity being reduced by participation in the program.

#### (i) Probability of children in the program target group following a life of crime

For the purposes of this study, it was conservatively estimated that 1% of children aged 10–14 years living in Melbourne displayed characteristics which would make them a potential candidate for the BBBS-M program. The task then was to estimate the proportion of these children who were likely, in the absence of an intervention, to become a criminal.

The overview by the Australian Attorney-General's Department of mentoring programs for youth at risk of offending [4] indicated that young people in the Australian programs displayed characteristics typical of young offenders, including poor family relationships, lack of appropriate role models, low socio-economic background, multiple problems including substance abuse, family violence, poor educational achievement and behavioural problems. Whilst the BBBS-M program is not geared specifically to young offenders or children at risk of offending, the profile of the Littles has close parallels. They comprise a seriously disadvantaged clientele who are often faced with multiple problems covering the gamut of conduct and behaviour problems, difficult family circumstances, violence and abuse and schooling problems. Many have multiple additional psychosocial risks including living in a foster family or residential care, exposure to conflict associated with parental custody disputes, having a sibling or parent with a disability, and economic adversity and poverty. Whilst the data was incomplete, a number of Littles were known to have been the subject of child protection court orders at the time of their referral.

Whilst it is not possible to predict individual propensity for adult criminality or substance abuse, many in the client group either currently displayed the characteristics or were from social and economic backgrounds that are common among adolescents who later pursue such high-risk behaviour. For the purposes of the modelling, it was assumed that half of the youths enrolled in the program were in the 'high-risk' category, i.e. equivalent to 0.5% of Melbourne children in the eligible age group.

#### (ii) Probability of high-risk behaviour being reduced by participation in the BBBS-M program

BBBS-M had not consistently collected outcome data, which enabled a comparison of the behaviour of program participants and non-participants (such as those in a waiting-list control group). Therefore, it was necessary to rely on published evidence to provide the link between participation in youth mentoring programs and consequent reductions in high-risk behaviours.

A number of studies have reported benefits of youth mentoring in reducing negative behaviours [10,19-21]. The Attorney-General's Department report [4] cites several programs where youth mentoring has been highly successful in reducing recidivism rates. Nevertheless, there is some conflict in the literature about the effectiveness of mentoring and, given the lack of evaluation studies with a proper control group, it is difficult to be definite about the likely reduction in high-risk behaviour amongst participants. The Australian report draws the same conclusion as other authors [29-31] that there is insufficient evidence to provide definitive proof that mentoring is effective, but it should be viewed as a promising strategy. It is for this reason that our research did not specify a probability of high-risk behaviour being reduced, but rather resorted to the use of threshold analysis which specified the number of cases needing to be averted for the BBBS-M program to break even.

## Results

The total cost of providing the BBBS-M mentoring service to currently matched youth in 2004 was AUD689,093, which equates to an annual cost of AUD 6,264 per each of the 110 young people currently in an active match. As at June 2000, there were an estimated 220,755 children aged 10–14 years resident in the Melbourne Statistical Division [32], which is the catchment for the BBBS-M program. If 1% of these children (2,208) were recruited to the program, and were involved in an active match for an average of three years, the total cost (with 5% discounting) of delivering the program over three years would be AUD 39.5 M.

Assuming that half of the youths enrolled in the program (1,104) are in the 'high-risk' category, the associated costs of juvenile and adult criminal behaviour would be AUD 3.3 billion (based on an average cost of AUD 3 M per youth).

The threshold analysis (Table 3) shows for any percentage reduction in prevalence, the reduced number of cases of high-risk youth expected, plus the associated cost savings. To 'break even' or pay for itself, the BBBS-Melbourne program would need to avert between 1–2% of cases of 'high-risk' youth out of the 1,104 modelled for Melbourne. A reduction of only 14/1104 cases or 1.3% would result in a cost savings of AUD 42 M which is more than the total cost of AUD 39.5 M of delivering the program for three years to the recruited cohort of 2,208 children. A reduction in prevalence of cases below this level does not infer that the program is not cost-effective, but simply means there is a positive cost associated with the intervention. With greater levels of effectiveness, the program becomes dominant in that it costs less than the amount it saves. If the benefits are assumed to start applying at age 18 years and are discounted at 5% back to age 10 years (the age of many children in the program) to be consistent with costs, a slightly higher number and proportion of cases (19 or 1.9%) would need to be averted.

**Table 3:** Threshold analysis

		**Undiscounted benefits**			**Discounted benefits**		
		
**Reduction in prevalence**	**Number of cases averted^a^**	**Potential cost savings^b^**	**Net benefits (costs, AUD)^c^**	**Incremental cost-effectiveness ratio (ICER, AUD)^d^**	**Potential cost savings^b^**	**Net benefits (costs, AUD)^c^**	**Incremental cost-effectiveness ratio (ICER, AUD)^d^**
10.0%	110	$330,000,000	$290,500,000	-$2,640,909	$212,740,000	$173,240,000	-$1,574,909
6.0%	66	$198,000,000	$158,500,000	-$2,401,515	$127,644,000	$88,144,000	-$1,335,515
5.0%	55	$165,000,000	$125,500,000	-$2,281,818	$106,370,000	$66,870,000	-$1,215,818
4.0%	44	$132,000,000	$92,500,000	-$2,102,273	$85,096,000	$45,596,000	-$1,036,273
3.0%	33	$99,000,000	$59,500,000	-$1,803,030	$63,822,000	$24,322,000	-$737,030
2.0%	22	$66,000,000	$26,500,000	-$1,204,545	$42,548,000	$3,048,000	-$138,545
1.8%	20	$60,000,000	$20,500,000	-$1,025,000	$38,680,000	-$820,000	$41,000
1.6%	18	$54,000,000	$14,500,000	-$805,556	$34,812,000	-$4,688,000	$260,444
1.4%	15	$45,000,000	$5,500,000	-$366,667	$29,010,000	-$10,490,000	$699,333
1.2%	13	$39,000,000	-$500,000	$38,462	$25,142,000	-$14,358,000	$1,104,462
1.0%	11	$33,000,000	-$6,500,000	$590,909	$21,274,000	-$18,226,000	$1,656,909

^a ^Refers to the number of cases averted as a result of the program. The total number of children in the program considered to be 'high-risk' cases is 1,104.

^b ^Refers to the lifetime costs of high-risk youth averted by reducing the prevalence. Savings equate to the number of cases averted × AUD 3 M (undiscounted) ($1.934 M discounted) per case

^c^Net benefit is derived by subtracting the costs of the BBBS-M program (AUD 39.5 M) for the cohort from the potential cost savings. A positive number means that the program saves more resources than what it costs, and conversely, a negative number means that the program costs more than what it saves.

^d^The ICER is determined by dividing the net benefits of the program by the numbers of cases of high-risk youth averted. A negative ratio denotes that the program is a dominant intervention in that it saves money and has extra benefits (defined as cases averted).

## Discussion

The analysis suggests that the BBBS-M program represents very good 'value for money' in that it offers the potential to provide long-term cost savings of much greater value than the costs of delivering the program. This is essentially because the program is relatively inexpensive to deliver, yet it affords the likely prospect of assisting young people at 'high-risk' of antisocial adult behaviours to avoid criminality and substance abuse, thereby saving the associated high costs to society.

The annual calculated cost of the BBBS-M program per match of AUD6,264 is reasonably close to the equivalent cost for BBBS-A reported by the Washington State Institute for Public Policy 2004 [12]. The American cost (which similarly includes volunteer time) was US4010, which equates to AUD 5,668 when historical conversion rates (AUD1.4135 = US1.00) are applied.

Whilst it is reasonable to conclude that the benefits of the program exceed its costs, the evaluation results should be considered indicative given both the lack of outcome data on the BBBS-M program's effectiveness, and the limited existing evidence of the value of its potential benefits. The economic modelling necessarily relies on a number of assumptions, which whilst conservative, require verification. Given the absence of more rigorous evidence, this economic evaluation is necessarily characterised by a number of uncertainties. These include the proportion of the age cohort meeting the eligibility criteria for the BBBS-M program, the cost estimates for saving a high-risk young person, the proportion of young people who without an intervention are likely to embark on a high-risk pathway, and in particular, the effectiveness of the BBBS-M program in diverting participants from such a lifetime pathway. The costs associated with operating the program are the subject of less uncertainty in that they have been developed by a 'bottom-up' costing approach. A number of variations in assumptions were tested and were found to have little impact on the overall conclusions (Table 4).

**Table 4:** Sensitivity results (benefits discounted)

**Reduction ** **in prevalence**	**Number ** **of cases** **averted^a^**	**Potential ** **cost** **savings^b^**	**Net benefits** **(costs AUD)^c^**	**Incremental ** **cost-effectiveness** **ratio ** **(ICER, AUD)^d^**
**Sensitivity 1: reduce value of saving high-risk youth to $1.673 M (base case $1.934 M)**				
2%	22	$42,548,000	$3,048,000	-$138,545
1%	11	$21,274,000	-$18,226,000	$1,656,909
**Sensitivity 2: increase value of saving high-risk youth to $2.264 M (base case $1.934 M)**				
2%	22	$49,808,000	$10,308,000	-$468,545
1%	11	$24,904,000	-$14,596,000	-$1,326,909
**Sensitivity 3: increase proportion of participants likely to embark on a high-risk pathway to 3% (base case 0.5%)**				
2%	132	$255,288,000	-$215,788,000	-$1,634,758
1%	66	$127,644,000	-$88,144,000	-$1,335,515

^a ^Refers to the number of cases averted as a result of the program. The total number of children in the program considered to be 'high-risk' cases is 1,104

^b ^Refers to the lifetime costs of high-risk youth averted by reducing the prevalence. Savings equate to the number of cases averted × AUD 1.934 M per case

^c ^Net benefit is derived by subtracting the costs of the BBBS-M program (AUD 39.5 M) for the cohort from the potential cost savings. A positive number means that the program saves more resources than what it costs, and conversely, a negative number means that the program costs more than what it saves.

^d ^The ICER is determined by dividing the net benefits of the program by the numbers of cases of high-risk youth averted. A negative ratio denotes that the program is a dominant intervention in that it saves money and has extra benefits (defined as cases averted).

The Cohen paper [26] was of key interest to our study in that it was answering a similar research question *"How many career criminals must be prevented before the program pays for itself?" *It is recognised that there are questions about the transferability of lifetime costs presented by Cohen from the USA to the Australian context. Cost structures are likely to differ (eg. market wage rates, the costs associated with the criminal justice system, or with drug treatment). The target population is also likely to be different. Nevertheless, these estimates are useful in providing some indicative data on the benefits which need to be forthcoming from the BBBS-M program to make it a worthwhile investment. Even if the cost of saving a high-risk youth was substantially reduced to a very conservative estimate of AUD 1 M (rather than the $3.3 M used in the analysis), the intervention would still break even when a not unrealistic 4% of cases (one in 25) was saved.

In adopting a comparison of children receiving the BBBS-M program with those not receiving the program, it is acknowledged that children in the latter group are likely to be in receipt of some other support services which have not been costed. Given that many BBBS Littles display problematic social behaviour, there is likely to be some involvement from youth justice or social care services.

Our analysis estimated that 1% of children in the target age group in Melbourne were likely to display characteristics which would make them a potential candidate for the BBBS-M program. This is a very conservative assumption, given data from the report on the mental health of young Australians [33]. The latter report estimates that 7.1% of children in the age range 4–17 years experience delinquent behaviour problems, 6.1% attention problems, 5.2% aggressive behaviour and 4.3% are withdrawn. All of these are typical behavioural problems which characterise the BBBS-M client group.

Whilst the BBBS-M mentoring program is not geared specifically to young offenders or children/adolescents at risk of offending, the profile of its youth participants parallels closely the characteristics typical of young offenders including parental drug or alcohol abuse, problems at school, poor family relationships, family violence, behavioural problems, low educational achievement and drug use [4,34]. Whilst it is impossible to be definite about the proportion of referred youth who might follow a path of juvenile crime or heavy drug use, it is reasonable to conclude that many in the client group display characteristics or come from a background typical of adolescents who pursue such high-risk behaviours.

Further research is required to estimate the true effectiveness and cost offsets of the intervention. The need to resort to threshold analysis as the form of economic evaluation was a consequence of the absence of outcome data for the BBBS-M program. A full cost-effectiveness analysis relies on the availability of both good costing data and measurable outcomes on the effectiveness of the program. Appropriate outcomes need to be identified and their measurement built-in to the ongoing data collection procedures of this and other non-government organisations providing essential social services.

## Conclusion

Despite these study limitations, even modest reductions in the prevalence rates of high-risk behaviour were shown to be sufficient to make the BBBS-M program a "dominant" intervention, (meaning it both saves money and provides extra benefits relative to the alternative of a 'do nothing' comparator). Nevertheless, whilst the program has been shown to "break-even" when it averts or "saves" around 2% of its high-risk youth participants, it may be that such a low proportion would not be acceptable to program management or funding bodies. However, focussing on the very small proportion of youth who may be saved from a high-risk lifetime (progressing from being a high-school drop-out, a major drug user to a career criminal) neglects the potential for other youth to be averted from lesser high-risk pathways (from being a high-school drop-out without the life of crime), thereby further adding to the net benefits of the program. It is fair to conclude that the BBBS-M program would appear to offer excellent "value-for-money", and represents a highly cost-effective use of public health or welfare resources.

## Competing interests

Author, Jane Fisher, was a member of the Board of Directors of BBBS-M from 2002 to 2007. The authors declare that they have no competing interests.

## Authors' contributions

MLM developed the study protocol, undertook the data collection and analysis and wrote the manuscript. JF made a substantial intellectual contribution to the conception and design of the study, and to the critical review of the manuscript.

## Pre-publication history

The pre-publication history for this paper can be accessed here:



## Supplementary Material

Additional file 1 **Annual costs of implementing the BBBS-M program and data sources.** The table shows the itemised costs of implementing the BBBS-M program and the sources from which the data was obtained. Click here for file
