# Supplementary material for: Are youth mentoring programs good value-for-money? An evaluation of the Big Brothers Big Sisters Melbourne Program
Source: BMC Public Health. 2009 Jan 30;9:41. doi: 10.1186/1471-2458-9-41 (PMC2640473; doi:10.1186/1471-2458-9-41)
Supplement: Additional file 1 — Annual costs of implementing the BBBS-M program and data sources. The table shows the itemised costs of implementing the BBBS-M program and the sources from which the data was obtained. [file 1471-2458-9-41-S1.doc]

**Additional files**

**Additional file 1**

**File format:** DOC

**Title:** Annual costs of implementing the BBBS-M program and data sources

**Description:** The table shows the itemised costs of implementing the BBBS-M program and the sources from which the data was obtained.

Additional file 1 Annual costs of implementing the BBBS-M program and data sources

| **Item** | **Definition** | **Unit cost**  **(AUD)** | **Total annual cost**  **(AUD)** | **Source of unit cost data** | **Assumption** |
| --- | --- | --- | --- | --- | --- |
| **Paid labour** | Management staff (per hour) | $28.59 | $79,099 | BBBS records | One full-time executive officer |
|  | Coordination (per hour) | $20.35 | $176,931 | BBBS records | A team leader/coordinator and 4 coordinator positions |
|  | Administration (per hour) | $14.71 | $40,548 | BBBS records | 1.4 EFT office manager/administrative positions |
|  | Salary on-costs |  | $44,487 | BBBS records | 15% to cover superannuation, WorkCover, leave |
|  | Accounting (per hour) | $30.00 | $1,200 | BBBS records | Contract ~ 3.3 hours per month; estimated rate |
|  | Auditor (per year) | $2,600 | $2,600 | BBBS records | Annual contract amount |
|  | IT support (per hour) | $30.00 | $2,700 | BBBS records | Contract ~ 7.5 hours per month; estimated rate |
| **Volunteer labour** | Mentors (per hour) | $16.00 | $238,848 | [4] | 4 hours: weekly for 1^st^ year matches; fortnightly matches 1-5 years; monthly -matches > 5 years. Estimated 14,928 volunteer hours annually. |
|  | Administrative support (per hour) | $14.71 | $3,060 | BBBS records | 4 hours per week |
|  | Client database support (per hour) | $43.00 | $1,032 | Gottliebsen Research & Skills Hub, 2004 | Estimate of 2 hours per month |
|  | Graphic design support (per year) | $1,800 | $1800 | Imputed | Pro bono support for annual report, newsletter etc |
|  | Book-keeping support (per month) | $180 | $2,160 | Imputed | Pro bono support. Estimate |
|  | Strategic planning facilitator (per year) | $2,000 | $2,000 | Imputed | Conduct of strategic planning exercises. Estimate |
|  | Legal services support (per hour) | $120.00 | $2,400 | BBBS records | Estimate of ~ 20 hours per annum |
|  | Accounting support (per hour) | $70.00 | $2,772 | BBBS records | Estimate of ~ 3.3 hours per month. Estimated difference between paid and market rate |
|  | IT support (per hour) | $70.00 | $6,300 | BBBS records | As above |
| **Accommodation** | Rental of premises (per month) | $1,111 | $13,333 | Imputed | Imputed market rental |
|  | Utility services (per year) | $4,000 | $4,000 | Estimate | Includes electricity, gas, water |
|  | Cleaning (per hour) | $15.00 | $3,120 | BBBS records | 4 hours per week |
|  | Security (per month) | $100 | $1,200 | Estimate |  |
|  | Paper recycling (per year) | $200 | $200 | BBBS records | One vehicle leased |
| **Transport** | Vehicle lease (per month) | $475 | $5,697 | BBBS records |  |
|  | Vehicle operating costs (per month) | $104 | $1,248 | BBBS records | Covers fuel, maintenance, insurance, toll fees |
|  | Private vehicle use (per km) | $0.59 | $24,233 | Royal Automobile Club of Victoria  www.racv.com.au | Reimbursement of use by staff (~3,600km) and Bigs (~37,320km). RACV rate for medium 2-3 litre vehicles.  Bigs – allowed 10 km travel each contact visit |
| **Other** | Telephone (per month) | $442 | $5,304 | BBBS records |  |
|  | Telephone calls by volunteers (per call) | $0.175 | $1,001 | Telstra | Assume one call per Big per week |
|  | Photocopier lease (per month) | $536 | $6,432 | BBBS records |  |
|  | Printing, toner, paper (per year) | $6,826 | $6,826 | BBBS records |  |
|  | Postage (per month) | $202 | $2,424 | BBBS records |  |
|  | Police checks (per check) | $12.30 | $738 | Victoria Police | 5 checks per month. Subsidised fee for volunteers |
|  | Psychological profiling (per profile) | $100 | $5,400 | BBBS-Sydney | By clinical psychologists. 54 checks per annum |
|  |  |  |  |  |  |
|  | **Total annual cost** |  | **$689,093** |  |  |
